# The effects of intra- and post-operative anaesthesia and analgesia choice on outcome after gastric cancer resection: a retrospective study

**DOI:** 10.18632/oncotarget.16724

**Published:** 2017-03-30

**Authors:** Yu Wang, Liping Wang, Hong Chen, Yang Xu, Xiaoyu Zheng, Guonian Wang

**Affiliations:** ^1^ Department of Anaesthesiology, Cancer Hospital of Harbin Medical University, Harbin, China

**Keywords:** anaesthesia, epidural and or general anaesthesia, patient-controlled analgesia, gastric cancer, overall survival

## Abstract

**Background:**

Epidural use can provide a better short-term outcome and protect patients from the postoperative development of tumour recurrence and metastases. In this study, we sought to assess the effects of intra- and postoperative anaesthesia and analgesia choice on outcome after gastric cancer resection, searched for evidence of interaction between intra-and postoperative epidural use and outcomes of gastric cancer patients.

**Methods:**

Four thousand two hundred and eighteen cases of gastric cancer were identified from the Records of Hospital Patients. Patients who received only general anesthesia (GA group) or epidural anesthesia combined with general anesthesia (EGA group), were administered patient-controlled intravenous or epidural analgesia for 72-120 hours postoperatively. Flatus time, length of stay in hospital, incidence of nausea and vomiting, and visual analogue scale (VAS ) scores were collected for evaluating the short-outcome of the patients. A Kaplan-Meier log-rank test was used for a univariable analysis, and Cox proportional hazards regressions were used for a multivariable analysis of the survival time in both groups.

**Results:**

The VAS scores and incidence of nausea and vomiting in the EGA group were lower than the GA group. There was a significant association between intra-and postoperative epidural use and improved survival.

**Conclusions:**

These results indicated that epidural anaesthesia combined with general anaesthesia and patient-controlled epidural analgesia may be associated with the improved overall survival in gastric cancer patients who underwent resection.

## INTRODUCTION

Gastric cancer is one of the most common malignant tumours of the digestive tract in China, and surgery remains the first-line treatment for gastric cancer patients. [1, 2] Even if optimal surgical techniques are used, tumour surgeries may release tumour cells into the lymphatic and vascular systems. Moreover, a large fraction of patients already harbour remote foci of tumour cells at the time of surgery; thus undetectable micro-metastases may already exist even in cases involving apparently localized disease. [3-5] Whether minimal residual disease [6] or released tumour cells result in clinically apparent disease depends largely on the balance between immune activity and the ability of tumour cells to invade, proliferate, and promote angiogenesis. [7] Surgery-stimulated immunosuppression,such as the decreased activity of natural killer (NK) cells and lymphocytes, may induce growth and metastasis of residual cancer cells, thereby leading to a worse prognosis. [8] The other clinical events, such as opioid analgesia acute pain, general aneasthetics, and transfusion are also recognized as immunosuppressive, and as a result, tumour recurrence and metastases may occur frequently. [9-14]

Epidural use has been shown to be associated with better analgesic effect, reducing flatus time, and length of hospital stay [15, 16], it can also decrease intra- and postoperative neuroendocrine stress responses, reduce opioid exposure that lead to immunosuppression, and relieve pain induced by surgery. [17, 18] These results lead to the hypothesis that epidural use may provide survival advantages in patients with several kinds of cancers. Recently, regional anaesthetic techniques have been demonstrated to be associated with better overall survival of several types of cancer, including breast cancer, hepatocellular carcinoma ,colorectal cancer. [19-22] Although widely believed to improve patient outcomes, results for survival after gastric cancer have been rarely reported. [23, 24] In this study, we sought to assess the interaction between intra-postoperative epidural analgesia and short/ long-term outcomes of gastric cancer.

## MATERIALS AND METHODS

### Patient identification and exclusion

**Figure 1:**
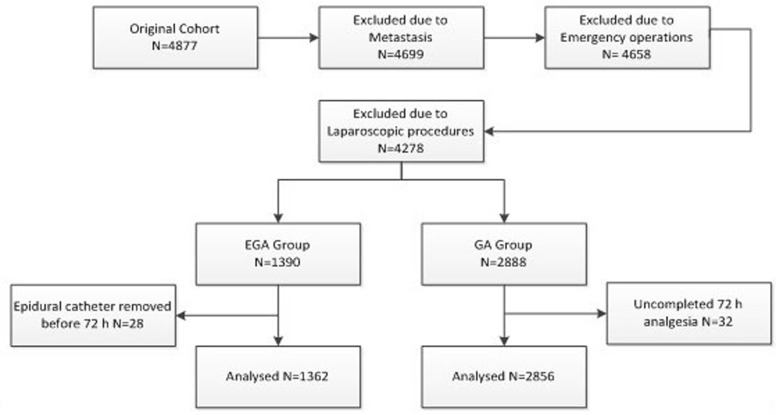
Patient identification and exclusion

After the study procedures were approved by the Ethics Committee of The Cancer Hospital of Harbin Medical University, 4218 gastric cancer cases were identified from the records of patients and patients admitted to the hospital for gastric cancer resection between 2008 and 2012 (Figure 1). Patients with metastasis, emergency operations and laparoscopic procedures were excluded. Patients who experienced anaesthesia and analgesia consisting with the following standard, and postoperative pathologies with gastric cancer were included. Medical records for all of the included patients were obtained, and the data were extracted by researchers who were not involved in the study or data analysis.

### Anaesthesia technique and grouping method

Between 2008 and 2012, the local departmental policy was to offer either epidural anaesthesia in combination with general anaesthesia or general anaesthesia alone. Patients who received only general anaesthesia (GA group) underwent anaesthesia induced with midazolam 0.05-0.15 mg/kg, 0.5 μg/kg fentanyl and 1-2.5mg/kg propofol. Propofol/sevoflurane and remifentanil/fentanyl were administered to maintain adequate anaesthesia depth during surgery. Patients received patient-controlled intravenous analgesia (PCIA) with 3 μg /ml fentanyl or 0.5 μg /ml sufentanil for 72-120 hours postoperatively. For patients who were given epidural anaesthesia (EGA group), a standard technique was used to insert a catheter into the epidural space of T8-T9 before induction of anaesthesia. Each of these patients was given a 3ml bolus of 1.33% lidocaine before induction of general anaesthesia. An infusion of 0.5% levobupivacaine or ropivacaine was administered to maintain an adequate anaesthetic concentration during surgery. In addition, patients received patient-controlled epidural analgesia ( PCEA) with 0.125% levobupivacaine combined with 2 μg /ml fentanyl or 0.2% ropivacaine combined with 2 μg /ml fentanyl were continued for 72-120 hours postoperatively.

### Indicator and data

The statuses of patients up to March 31, 2015 were determined from medical records, and causes of death were recorded. Patients who died due to causes other than gastric cancer were censored. We obtained the following information: demographic data; cancer stage; American Society of Anaesthesiologists (ASA) grade; duration of surgery; axillary temperature after surgery; degree of differentiation; transfusion, preoperative or postoperative adjuvant chemotherapy, and/or radiation therapy; flatus time; length of stay; days of analgesia; visual analogue scale (VAS) scores were received. Cancer stage was assessed based on the 7^th^ edition of American Joint Committee on Cancer (AJCC) Cancer Staging Manual. The degrees of differentiation included well differentiated, other/unknown differentiated, moderate differentiated, and poorly differentiated. Survival time was measured from the date of gastrectomy to death or to the last followed-up before March 31, 2015.

### Statistical approach

We recorded study data in an Excel spread sheet that was then imported into SAS software for analysis (version9.13, SAS Institute Inc, Cary, NC). The GA and EGA groups were compared with respect to all available potential confounders using Pearson Wilcoxon rank sum test, the chi-squared test, and Fisher’s exact probability test, as appropriate. The survival time for the two groups was assessed using Kaplan–Meier log-rank test for the univariable analysis, and Cox proportional hazards regression for the multivariable analysis. Factors considered and retained in the multivariable models include age, cancer stage, degree of differentiation, duration of surgery, blood loss, transfusion and history of chemotherapy and radiotherapy. Associations with *P* < 0.05 were deemed statistically significant.

## RESULTS

### Patient characteristics

Using the inclusion and exclusion criteria described above, we identified a cohort of 4218 patients (Figure 1), 67.7% of whom (*n* = 2856) were in the GA group, and 32.3% of whom (*n* = 1362) in the EGA group. The median follow-up times for the GA and EGA group were 42.5 months and 38.7 months. The groups exhibited no differences in age, height, weight, duration of surgery, gender, smoking history, alcoholism, hypertension history, ischaemic cardiomyopathy, diabetes, ASA grade or temperature after surgery. Moreover, no differences in cancer stage or degree of differentiation were observed between the two groups (Table 1).

**Table 1:** Baseline and surgical characteristics

Characteristics	GA group (2856)	EGA group (1362)	*P* value
Age (y)	58±8.3	58±9.5	0.341
Height(cm)	170±7.4	168±8.2	0.124
Weight(kg)	63.5±9.4	61.3±8.7	0.264
Duration of surgery(h)	3.25 (3.00,3.75)	3.25 (3.00,3.50)	0.055
Gender(male)	2226 (77.94%)	1058 (77.68%)	0.848
Smoking(yes)	1440 (50.42%)	714(52.42%)	0.224
Alcoholism(yes)	1244 (43.56%)	576 (42.29%)	0.437
Hypertension(yes)	306 (10.71%)	168 (12.33%)	0.119
Ischaemic cardiomyopathy(yes)	174 (6.09%)	72 (5.29%)	0.296
Diabetes(yes)	30(1.05%)	12 (0.88%)	0.605
ASA 1	324 (11.34%)	178 (13.07%)	0.345
2	2376 (83.19%)	1142 (83.85%)	
3	156 (5.46%)	42(3.08%)	
Cancer stage I	816 (28.57%)	402 (29.52%)	0.235
II	132(4.62%)	48(3.52%)	
III	1908 (66.81%)	912 (66.96%)	
Degree of differentiation 1	1472(51.45%)	723 (53.08%)	0.088
2	1194 (41.81%)	528 (38.77%)	
3	78(2.73%)	48 (3.52%)	
4	112 (3.92%)	54 (3.96%)	
Temperature (°C)	33.2±1.8	32.9±1.5	0.425

Abbreviations: EGA, epidural anaesthesia combined with general anaesthesia group; GA, general anaesthesia group; ASA, American Society of Anaesthesiologists.

Degrees of differentiation: Degree1, poorly differentiated; Degree2, moderately differentiated; Degree3, well differentiated; Degree 4, other/unknown differentiated.

Cancer stages: Stage I-T1, N0, M0/T2, N0, M0/T1, N1, M0; Stage II-T3, N0, M0/T4a, N1, M0/T3, N1, M0/T2, N2, M0/T1, N3, M0; Stage III-T2, N3, M0/T3, N2, M0/T3, N3, M0/T4a, N2, M0/T4a, N3, M0/any T4b, any N, M0; Stage IV, any T, any N, M1..

### Association between epidural use and short-outcome

Comparisons between the EGA and GA groups with respect to flatus time, length of hospital stay, incidence of nausea and vomiting, days of analgesia, and VAS scores are presented in Table 2. The EGA group had lower VAS scores (*P* < 0.01) and incidence of nausea and vomiting (*P* < 0.05) in the postoperative three days. Perioperative epidural use showed no associations with other short-term outcome variables, such as flatus time, days of analgesia and length of stay.

**Table 2:** Short-term outcomes according to type of anaesthesia and analgesia

**Characteristics**	**GA group (2856)**	**EGA group (1362)**	***P* value**
Flatus time (days)	4 (3,5)	4 (3,5)	0.427
Length of stay (days)	19 (17,22)	18 (17,21)	0.123
Nausea and vomiting	505 (17.68%)	228 (16.74%)	<0.05*
Days of analgesia(days)	4 (3,5)	4 (3,5)	0.385
VAS scores			
POD 1	3 (2,5)	2 (1,4) *	<0.001*
POD 2	2 (1,4)	1 (0,3) *	<0.001*
POD 3	1 (0,3)	1 (0,2) *	<0.001*

**Abbreviations: VAS**=Visual analogue scale (VAS) scores, **POD**=Postoperative day

### Association between epidural use and long-term overall survival

**Figure 2:**
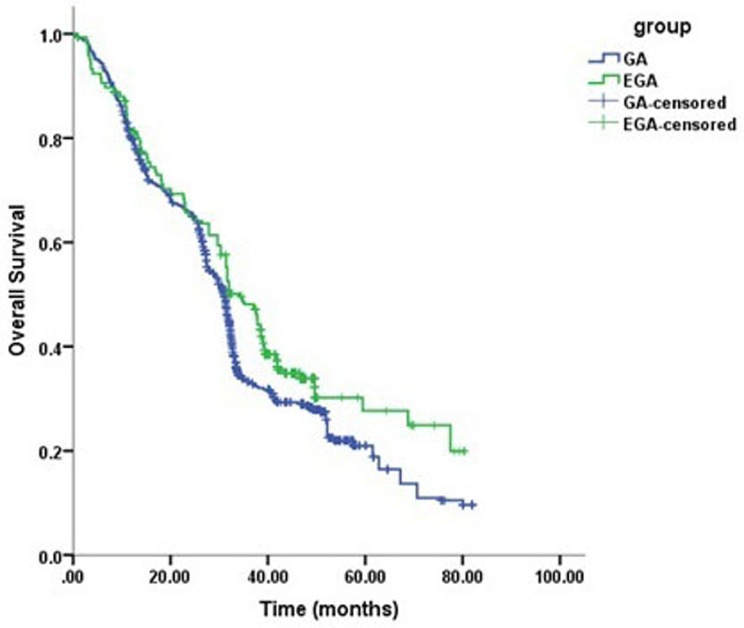
Kaplan–Meier survival curves for patients with and without epidural use (univariate *P* < 0.0001*)

The mean survival times in the GA and EGA groups were 35.1 months and 40.2 months, respectively. Perioperative epidural use was associated with overall survival (*P* < 0.0001, long-rank test), with an estimated hazard ratio (HR) of 0.65 (95% confidence interval [CI], 0.58-0.73) in the univariable analysis (Table 3).Cancer stage (HR = 1.41, 95% CI 1.29-1.45, *P* < 0.0001) and degree of differentiation (HR = 0.67, 95% CI 0.61-0.74, *P* < 0.0001) were also associated with overall survival. In the multivariable Cox model that considered only the statistical effect of epidural use (Model 1, Table 4), cancer stage, degree of differentiation were associated with epidural use, which exhibited an adjusted estimated HR of 0.70 (95% CI 0.63-0.77, *P* < 0.0001). Kaplan–Meier estimates of survival as a function of postoperative time for the two groups are provided in Figure 2. The resulting curves differed significantly ( *P* < 0.0001, log-rank test).

**Table 3:** Univariate associations with survival

**Factor**	***P* value**	**H R(95%CI)**
Blood transfusion (yes vs. no)	<0.0001*	0.61(0.49-0.78)
Cancer stage (higher vs. lower)	<0.0001*	1.41(1.29-1.45)
Degree of differentiation (higher vs. lower)	<0.0001*	0.67(0.61-0.74)
Chemotherapy or radiation therapy (yes vs. no)	0.486	0.97(0.87-1.07)
Epidural use (EGA vs. GA)	<0.0001*	0.65(0.58-0.73)
Age (≥65y vs. <65y)	0.439	1.05(0.93-1.18)

**Table 4:** Multivariate associations with survival: Cox multivariate Model 1, statistical effects only

Factor	*P* value	HR(95%CI)
Blood transfusion (yes vs. no)	<0.0012*	0.68(0.53-0.86)
Cancer stage (higher vs. lower)	<0.0001*	1.35(1.28-1.43)
Degree of differentiation (higher vs. lower)	<0.0001*	0.69(0.62-0.76)
Epidural use (EGA vs. GA)	<0.0001*	0.70(0.63-0.77)

## DISCUSSION

The main findings of this retrospective study were as follows: Our results suggest an early and sustained beneficial effect of epidural use on tumour-related mortality after gastric cancer. Firstly, in our study, the EGA group had lower VAS scores on the postoperative three days. The results were consistent with that of a recently study, it found epidural use was associate with less number of additional doses of analgesics, and provided better analgesic effect, [25] both the two studies found no difference in length of hospital stay between the epidural and non-epidural use groups. In our study, there were no differences in flatus time between the two groups, while in the above mentioned study, the first time of flatus was earlier in the epidural group. The incidence of postoperative nausea and vomiting was higher in the GA group in our study, however, in the above mentioned study, there were no differences between the two groups , the reason may be that the prior study collected patients underwent laparoscopic distal gastrectomy, but we collected patients with all kinds of gastrectomy.

Secondly, there was a significant association between perioperative epidural use and improved survival. We identified associations between cancer stage, degree of differentiation and overall survival after gastric cancer resection, the findings were consistent with those of prior observational studies that evaluated other types of cancers, such as ovarian, [26] breast, [27]colorectal [28] and gastric cancer. [23, 24] We also found that epidural use can improve the long-term outcome of patients with gastric cancer, which was negative in one of the gastric cancer studies, [23] the author collected the patients who had been over 66 years old, but in our study, the patients’ average age was 58 years old, and it has been found that epidural use was associated with reduced gastric cancer overall survival of younger patients, but not in older patients. [24] Our results were consistent with a study on gastro-esophageal cancer, it demonstrated that postoperative epidural analgesia can increase time to cancer recurrence and overall survival. [29] As mentioned previously, [23] the overall median survival durations were 28.1 months for the epidural group and 27.4 months for the non-epidural group, but in our study, the median survival durations for the EGA and GA groups were 40.2 and 35.1 months, respectively, that can also be explained by differences in patient populations, surgical technique, and time of admission.(our data provided greater age range and later admission time)

Tumour surgeries may release tumour cells into the lymphatic and vascular systems, whether released or harboured tumour cells result in clinically apparent disease depends largely on the balance between immune activity and the ability of tumor cells to invade, proliferate, and promote angiogenesis. Since immune surveillance is the first indicator for preventing cancer metastasis, immunesuppression may decrease the defensive barrier against tumour cells. [30] Clinical events that may lead to altered immune response after surgical trauma include tissue injury, pain, general anaesthesia, blood transfusion, and opioid drugs . [31-33] These clinical events stimulate activation of the hypothalamic-pituitary-adrenal (HPA) axis and the sympathetic nervous system (SNS) during the perioperative period. [34]The activation of multiple biological cascades leads to postoperative immunosuppression, which affects both humoral and cell-mediated responses. [10, 11] Then it lead to the suppression of NK cells and cytotoxic ( CTLs), in association with a decrease in interleukin-12 (IL-12), tumour necrosis factor-α (TNF-α), and interferon-γ (IFN-γ). [35] In our study, the EGA group had lower VAS scores on the postoperative three days, the immune function of patients in the EGA group may be less influenced , that may be the reason of better outcome in the EGA group.

The acute and chronic administration of opioids also inhibits components of cellar and humoral immune function from suppressing NKcell activity. [36-38]It has been proved that μ-opioid receptor agonists transactivate pro-oncogenic vascular endothelial growth factors (VEGF) and epidermal growth factor (EGF) receptors resulting in enhanced tumour growth (reversed by opioid receptor antagonism). [39] Epidural anaesthesia can decrease intra- and postoperative neuro-endocrine stress responses, [40-42] reduce opioid exposure [29, 33, 43] that leads to immunosuppression, then it may improve the outcome of cancer patients. It is a pity that we couldn’t collect the total volumes of opioid drugs or rescue analgesics, so the hypothesis of epidural use improved overall survival through less opioid exposure was not confirmed in our study. On the other hand, there were some studies suggest that epidural anaesthesia could not protect patients from immunosuppression in upper abdominal surgery, [44, 45] so the hypothesis that epidural use can improve overall survival of gastric cancer is still controversial, and the interaction between epidural use and outcomes of gastric cancer is influenced by several kinds of mechanisms.

We found an association between perioperative blood transfusion and an increased hazard for mortality which is consistent with the increasing recognized negative effects of transfusion. Kenneth *et al* [28] and Glance *et al* [46] found an association between blood transfusion and an increased risk of death, in gastric cancer and non cardiac surgery. This may because blood transfusion can induce suppressed immune functions. Alternative explanations are that the surgery on patients with comorbidities (anemia of chronic disease), or that surgery on patients requiring transfusion was more complex imposing greater inflammatory load. [29]

Our study had certain limitations. Firstly, the clinical data, such as detailed surgical techniques, special drugs administered, perioperative opioid use which induced biases, could not be collected. Secondly, we were unable to determine certain indices that reflected immune systemic functions, therefore, we should design prospective studies to clearly elucidate the mechanisms underlying our results, NK cell activity and markers of immunological function, such as cytokines and cortisol will be measured.

In conclusion, this study suggests that epidural anaesthesia combined with general anaesthesia and patient-controlled epidural analgesia may provide better analgesic effect and be associated with the improved overall survival in gastric cancer patients who underwent resection.
